# Editorial: Endocrine insights into heart disease

**DOI:** 10.3389/fendo.2025.1664575

**Published:** 2025-07-25

**Authors:** Rogério Faustino Ribeiro Júnior, Daigo Sawaki, Li F. Chan, Gabor Czibik

**Affiliations:** ^1^ Department of Physiological Sciences, Federal University of Espirito Santo, Vitoria, ES, Brazil; ^2^ Department of Clinical Pharmacology and Cardiology, Jichi Medical University, Shimotsuke, Japan; ^3^ Centre for Endocrinology, William Harvey Research Institute, Barts and the London School of Medicine and Dentistry, Queen Mary University of London, London, United Kingdom

**Keywords:** cardiovascular disease, classic hormones and cardiovascular health, body composition and metabolism, effect of blood glucose +/- lipoprotein fractions on cardiovascular pathologies, miscellaneous clues

Apart from familial cardiomyopathies evolving based on disease-causing mutations of myocardial proteins, cardiovascular diseases do not develop in isolation. Common causes of and composite effects in cardiovascular disease tend to be more elusive. Some inducers include unhealthy diets promoting atherosclerosis or hypertension and age as well as sedentary lifestyle that are recognised determinants of cardiovascular disease. Increased incidence and prevalence of cardiovascular disease in patients with chronic liver or kidney disease underscore the importance of interdependent organ-organ interactions in health and disease. Sustained overactivation of the sympathico-adrenergic or renin-angiotensin-aldosterone systems (RAAS) straining the heart or vasculature illustrate classic endocrine influences over the cardiovascular system. These examples notwithstanding, relatively little is known about how dysfunction of one organ begets dysfunction in the cardiovascular system (1). Due to the reductionistic nature of scientific studies, there is a long way to the discovery of how seemingly disparate alterations impair cardiovascular health. This is particularly so when classic physiology does not come to the aid of our investigations.

There is much to be learned about an organism composed of symbiotic cells fated to live and die together. Questions include, but are not limited to how adverse influences work? Does a singular abnormality emerge as a loss-of-function of physiological activity (e.g. loss of kidney function altering extracellular volume or ion composition) or gain-of-function to induce detrimental changes (e.g., secretion of factors promoting organ damage)? How is the damage signal conveyed: via secretion, through bodily fluids, such as blood, the neural system or some other way? Which cells are responsible, and which ones are affected? How does the effect manifest? Are molecular effects mediated through receptors expressed by certain cell types or using other channels of cellular communication? Can we detect cardiovascular diseases from the blood? Are there easily measurable signs before a crippling disorder develops or is at a still reversible stage?

In line with these questions (to some of which we already have answers) there is a considerable clinical interest in discovering predictors (precede the condition, signifying its coming) or markers (simultaneous with the condition, and incrementally indicates degree of severity) of cardiovascular disease. Criteria for good disease predictors and markers include that they draw attention to a specific condition to be prevented or stopped with great accuracy, i.e., with negligible chance for false negative and/or positive cases. By replacing or reducing the need for expensive equipment and/or specialised expertise, these diagnostic tools are meant to be simpler, and more cost-effective than traditional diagnosis. Functionally a good predictor/marker may be an epiphenomenon, a change that develops contemporaneously with the condition, but with no causal effect on the condition *per se*. It could be for example a product with a minor function co-secreted in stochiometric quantity with a more important factor (i.e., C-peptide co-secreted with insulin). The other kind of predictor/marker is intricately involved in the pathogenesis of the condition (such as insulin in glucose homeostasis and anabolism), without which the condition would develop very differently (type I and II diabetes mellitus). With regard to the diagnosis alone, it makes no difference whether the predictor/marker is pathogenically involved or not, as long as the condition is precisely reported. Research aiming to identify easily measurable, affordable disease predictors/markers could be especially useful for screening masses of people. This is a very important practical consideration, because in our overburdened healthcare systems, people, especially the underprivileged, are at a disadvantage to access diagnosis. A pathogenic involvement of predictors/markers is a bonus, as increased mechanistic understanding may be exploited therapeutically in the long run. For example, increased circulating levels of catecholamines and angiotensin II led to the development of new classes of medicines. Today, the countless human lives saved with beta blockers (2) and RAAS inhibitors (3) exemplify the potential of therapeutically targeting circulating messengers of harm.

Our goal with the call “*Endocrine insights into heart disease*” was to attract work to help connect the dots on a landscape spanning from primary abnormalities, through their responses down to cardiovascular disease. The exciting studies in this Research Topic cover an extended range of endocrine abnormalities, as briefly outlined below.

## Classic hormones and cardiovascular health

Some of the papers offer novel insights into how imbalance of classic hormones may promote distinct cardiovascular conditions. Specifically, Liu et al. explored whether thyroid hormone replacement reverses diastolic dysfunction in adult patients with subclinical hypothyroidism. Whilst in this systematic review all 17 studies included reported normalised serum thyroid-stimulating hormone levels after levothyroxine treatment (n=568 patients), diastolic dysfunction was only partially rescued (e.g., E/A improved, but E/é did not). The authors admitted some study limitations (i.e., in sampling, inter-study variability in TSH values, limited observation times as well as unreported parameters with potential impact on diastolic function), which could be eliminated in a well-designed, large-scale randomised controlled study. do Val Lima et al. reported impaired myocardial contractility and oxidative phosphorylation in testosterone-deficient, orchidectomised rats. Intriguingly, mitochondrial dysfunction was restricted to interfibrillar mitochondria, i.e., those in charge of feeding sarcomeres with ATP, which could explain the link to contractile deficit. Myocardial effects of orchidectomy were not limited to mitochondrial bioenergetics, but also induced NADPH oxidase, a source of reactive oxygen species, whilst reduced expression of cellular antioxidant enzymes. Testosterone resupplementation rescued abnormalities. These observations support a unique sex-dependent role for testosterone in cardiac function, suggesting important clinical implications in patients undergoing castration due to disease or gender reassignment surgery. It will be interesting to see how and why testosterone controls interfibrillar but not subsarcolemmal mitochondria. Next, based upon the prothrombotic state observed in Cushing’s syndrome, Brosolo et al. hypothesized that cortisol interferes with haemostasis in patients with essential hypertension. Consistent with the hypothesis, plasma samples showed higher D-dimer, prothrombin fragments 1 + 2 and von Willebrand factor levels across the upper tertile of cortisol and cortisol response to dexamethasone overnight suppression. Multivariate regression analysis established a positive relationship of the above coagulation factors to both cortisol and cortisol response to dexamethasone overnight suppression, independent of age, body mass index, blood pressure, and renal function. By identifying glucocorticoid-mediated haemostatic propensity as a surrogate mechanism to cause organ damage, essential hypertension seems to be more than its classic rheological consequences alone. Watanabe et al. studied fibroblast growth factor 23 (FGF23) and RAAS activity in C57BL6/j mice subjected to surgical pressure overload induced by transverse aortic constriction (TAC) vs. sham surgery. Sequential analysis revealed that cardiac FGF23 transcript but not circulating FGF23 levels increased in the early phase of pressure overload. By contrast, cardiac angiotensin-converting enzyme (ACE) activity and serum aldosterone levels increased in parallel with pressure overload. In addition to controlling left ventricular hypertrophy and RAAS activity, ACE inhibition with enalapril normalised cardiac FGF23 transcript levels. Liu et al. explored whether increased circulating Klotho levels increase or decrease cardiovascular risk. To explore this, they used patient data with cardiovascular disease from the National Health and Nutrition Examination database (NHANES; n=1905 patients). The authors found a U-shaped curve in terms of both all-cause and cardiovascular mortality, suggesting that an optimal Klotho dose may be required for survival. Finally, considering that α-Klotho (a member of the Klotho family) cleaves and activates FGF23, it is intriguing to speculate whether α-Klotho acts via FGF23 or any independent substrate (4).

## Body composition and metabolism

Another important area revolves around body composition and metabolism. This is most timely due to the epidemic proportions obesity reaches, affecting our society in complex, elusive but predominantly bad ways. The effect is not simply incremental, however, i.e., the more obese the worse. The obesity paradox indicates that obese patients show improved outcomes in myocardial infarction, stroke, or heart failure (5), despite obesity considered as a major risk factor for developing the same diseases. The subject remains highly contentious, hence more data is needed to shed light on this curious phenomenon. Wang et al. contributed to this debate with a systematic review investigating mortality after intracerebral haemorrhage and mortality. They found that in all 10 studies that met the inclusion criteria, obesity was protective, with 8 studies being statistically significant, whilst in 6 studies obesity associated with significantly lower short- and long-term mortality. Apart from the mass effect, subtleties of adipose tissue accumulation are illustrated by the subsequent two studies. First, Wu et al. studied the relationship between regional fat distribution and risk of developing atrial fibrillation. This large-scale, prospective study involved nearly half a million patients with evaluation of total and regional adipose tissue percentage along with incident atrial fibrillation. Over a follow-up of more than a decade almost 30000 patients developed atrial fibrillation. Curiously, fat mass, particularly that of the legs, associated with a reduced risk of developing atrial fibrillation. Second, Wang et al. explored the relationship between the volume of epicardial adipose tissue and premature ventricular complexes (PVCs). This retrospective study involving consecutive patients with PVC vs. matched control subjects found that larger epicardial adipose tissue mass was positively correlated with PVCs, and associated burden. Larger epicardial adipose tissue was observed in male patients with PVCs, greater body mass (BMI ≧24 kg/m (2)), diabetes mellitus, and impaired left ventricular relaxation. Therefore, epicardial adiposity, an adipose depot embracing the pumping heart, seems to extend myocardial predisposition to arrhythmias other than atrial fibrillation (6), probably through an arrhythmogenic secretome. Next, Cooper et al. was puzzled by the reasons for heterogeneous low-density lipoprotein (LDL)-cholesterol response to ketogenic diets. To study this, they observed lean, healthy premenopausal women with controlled periods of ketogenic and carbohydrate-containing regimes. Reintroduction of carbohydrates to ketogenic diet made notable hormonal (i.e., increased free T3 levels) and metabolic changes (i.e., increased fat mass, insulin levels). Amongst these, plasma LDL-cholesterol levels dropped when ketosis was interrupted by dietary carbohydrates. Intriguingly, LDL-cholesterol changes only correlated with body composition metrics, thyroid hormones, but not other metabolic parameters. The authors concluded that consistent with the increased lipid turnover to meet energy demands, lean individuals exposed to ketogenic diet were more susceptible to increases in circulating LDL-cholesterol levels. Beside LDL-cholesterol levels, insulin resistance and inflammation are other important risk factors for coronary heart disease. Dong et al. explored the utility of novel biomarkers of insulin resistance (triglyceride-glucose index: TyG), and inflammation (system immune-inflammation index: SII; & systemic inflammation response index: SIRI) in coronary heart disease among patients with non-alcoholic fatty liver disease (NAFLD). Besides established factors (i.e., triglyceride, LDL-cholesterol & neutrophiles promote, whilst HDL-cholesterol protect against coronary heart disease), logistic analysis suggested that TyG, SII and SIRI were all independent risk factors for coronary heart disease in NAFLD patients. Further analysis indicated that these innovative biomarkers, individually and especially combined, have high predictive value, therefore may aid diagnostic efforts. Our quest for the future will be to uncover the molecular underpinnings behind these exciting observations.

## Effect of blood glucose +/- lipoprotein fractions on cardiovascular pathologies

Managing blood glucose is essential for reducing cardiovascular risks, but the intricate relationship between glycemic control and cardiovascular outcomes reveals deeper complexity, particularly involving different lipoprotein fractions. In particular, uncontrolled lipoproteins, through their varying biological effects, have been proposed to underlie divergent cardiovascular outcomes despite close glucose control. Studies highlight the distinct impacts of remnant- versus LDL-cholesterol in hypertension risk, demonstrate how apolipoprotein B mediates inflammation-related coronary heart disease, and emphasize the differential effectiveness of hypoglycemic agents in slowing carotid artery thickening, an early marker of cardiovascular disease. These observations underscore the necessity to adopt a more nuanced approach beyond standard blood glucose control and take specific lipid profiles and inflammatory markers into consideration in therapeutic decisions for cardiovascular disease.

Chen et al. took a deep dive into how SGLT2 inhibitors, originally designed to lower blood sugar in diabetes, protect patients with chronic kidney disease (CKD) from severe cardiovascular events. Remarkably, their analysis revealed that these medications significantly reduce hospital admissions for heart failure, the risks of heart-related and overall mortality. Even more reassuringly, these benefits did not come with increased rate of serious side-effects or urinary tract infections, though they did see an uptick in reproductive tract infections. This is invaluable knowledge for managing patients experiencing a combination of diabetes, kidney and heart disease. Shi et al. ventured beyond traditional markers like LDL-cholesterol, often tagged as "bad cholesterol," and highlighted the role of remnant cholesterol in hypertension. Their research underscored that higher levels of remnant cholesterol independently correlated with increased blood pressure—even in people whose LDL-cholesterol levels were within recommended limits. This finding challenges the traditional cholesterol paradigm, suggesting that clinicians should consider monitoring remnant cholesterol to more effectively identify and manage individuals at increased risk of hypertension and related cardiovascular diseases.

Taking a closer look at the inflammation-cholesterol nexus, Yang et al. investigated the roles of interleukin-1 receptor antagonist (IL-1Ra), a key player in inflammation, and apolipoprotein B (apoB), a critical component of LDL particles. Their groundbreaking study revealed that genetically higher IL-1Ra levels increased the risk of coronary heart disease. However, this risk dramatically reversed once apoB was considered, indicating a role for apoB as a pivotal mediator in inflammation-driven cardiovascular disease. This discovery not only enhances our understanding of heart disease but also offers a promising target for potential treatments aiming to tackle both cholesterol and inflammation simultaneously. Finally, Lv et al. offered valuable, practical insights by comparing customary diabetes medications and their long-term effects on carotid intima-media thickness (cIMT), a critical marker of early artery stiffening and atherosclerosis. Their extensive network meta-analysis identified exenatide, alogliptin, and metformin as particularly effective choices to slow cIMT progression. This indicates that these drugs may carry unique cardiovascular protective properties beyond their blood sugar-lowering effects, providing clinicians with options for a more holistic approach to cardiovascular care.

Collectively, these studies reveal a new level of complexity: managing blood glucose is not equally effective across different aspects of cardiovascular health, largely because different lipoprotein fractions may undo any good achieved through prudent glucose control. Understanding these interactions can empower healthcare providers with more precise, individualized strategies to combat cardiovascular diseases linked with diabetes and metabolic conditions, ultimately improving patient outcomes and quality of life.

## Miscellaneous clues

In this Editorial, we expose fascinating new insights revealed by recent research, exploring how hormones and other endocrine actors impact cardiovascular health. These studies help us better understand cardiovascular diseases, bringing us a step closer to easily accessible, early diagnosis and more effective treatments for improved patient care.

Tang et al. explored whether elevated serum levels of uric acid, the end-product of purine catabolism, challenge right ventricles of patients with connective tissue diseases. Using advanced cardiac imaging techniques, this study points out that higher serum uric acid levels are strongly linked to impaired right-sided heart function. This discovery underscores the importance of early diagnosis, and subsequent appropriate management of this condition, whilst providing mechanistic clues towards disturbed purine handling. Xu et al. shed light on an intriguing link between uterine fibroids and high blood pressure. Using a clever genetic analysis method, they established a direct connection between these benign tumors and elevated systolic blood pressure. This finding is particularly important for women's health, emphasizing the need for careful blood pressure monitoring in women with fibroids, especially during pregnancy. These findings also open the door to potential treatments that tackle uterine fibroids not just for their direct symptoms, but also as a means to control blood pressure.

Zou et al. used extensive hospital records to discover a link between abnormal serum osmolality (a measure of body fluid balance) and early mortality in heart failure patients. They found that, in a U-shape, both very low and very high levels increase the risk of death within the first month after hospitalization. This insight underscores how closely fluid and electrolyte management are tied to patient survival in acute heart conditions. Finally, Zhang et al. (2024) explored how the platelet-to-HDL cholesterol ratio may predict cardiovascular and mental health. Their study demonstrates a clear positive relationship between platelet-to-HDL cholesterol ratio levels, depression, and cardiovascular mortality. This finding suggests that platelet-to-HDL cholesterol ratio could serve as a simple and effective screening tool for depression-associated heart risks, ultimately improving early intervention efforts and patient outcomes.

Taken together, these studies offer crucial insights into the ways hormones and other endocrine signals affect our cardiovascular and overall health. By deepening our understanding of these mechanisms, we open new paths toward better diagnostics, targeted therapies, and ultimately, improved quality of life for patients battling cardiovascular diseases.
